# Recognition and control of *Mycobacterium tuberculosis*-infected cells: from basics to the clinic: a NIAID/WGNV workshop report 2023

**DOI:** 10.3389/ftubr.2023.1303505

**Published:** 2023-11-15

**Authors:** Carly Young, Mbali N. Mkhonza, Paul Ogongo

**Affiliations:** ^1^South African Tuberculosis Vaccine Initiative, Faculty of Health Sciences, Institute of Infectious Diseases and Molecular Medicine, University of Cape Town, Cape Town, South Africa; ^2^Division of Immunology, Faculty of Medicine and Health Sciences, Biomedical Research Institute, Stellenbosch University, Cape Town, South Africa; ^3^Stop TB Partnership Working Group on New TB Vaccines, New York, NY, United States; ^4^Division of Experimental Medicine, School of Medicine, University of California, San Francisco, San Francisco, CA, United States

**Keywords:** *Mycobacterium tuberculosis*, recognition, control, infected cell, immunity, vaccines, antigens

## Abstract

Vaccination is crucial for the control of tuberculosis (TB), and safe, more effective, and accessible vaccines against *Mycobacterium tuberculosis (Mtb)* infection are critically needed to achieve TB control milestones envisioned in the End TB Strategy. TB vaccine research and development faces numerous challenges including, but not limited to, insufficient knowledge of the most informative antigens to prioritize as potential vaccine candidates, lack of defined correlates of protection, and incomplete knowledge of anatomical and cellular locations of the *Mtb*-infected cell *in vivo*, among others. To take stock of the progress, challenges, and opportunities in TB vaccine R&D, the Stop TB Partnership Working Group on New TB Vaccines (WGNV), in partnership with the National Institute of Allergy and Infectious Diseases (NIAID) cohosted a two-day virtual workshop on 13–14 June 2023 with experts from all over the world. In this report, we summarize key themes and discussions from the meeting, highlighting progress and gaps in the TB vaccine research.

## 1 Introduction

Under the theme “Recognition and control of *Mycobacterium tuberculosis* (*Mtb)*-infected cells: from basics to the clinic,” the objectives were to: (i) Elucidate the mechanisms by which the immune system recognizes *Mtb*-infected cell, (ii) Explore when, where, how, and the degree to which recognition can lead to control of the intracellular microbe, (iii) Discuss the translational implications of current developments in the field, and (iv) Foster discussions to address gaps in our understanding of the immune response to *Mtb*, to enable identification of correlates of protection and development of novel interventions like new TB vaccines.

### 1.1 Antigens displayed by the infected cell

The first session explored mechanisms by which the host immune system recognizes the *Mtb*-infected cell. Paul Ogongo (University of California San Francisco) discussed the evidence for disease-specific antigens. Ogongo shared a report by Musvosvi et al. (1), which identified antigenic peptides targeted by T-cell receptor similarity groups associated with control or progression, demonstrating that distinct *Mtb* antigens are associated with infection outcomes. In a second report by Meier et al. (2) cytokine responses to distinct *Mtb* antigens differentiated TB progressors from non-progressors 2 years prior to TB progression. Finally, Ogongo described his unpublished work in the Ernst Lab, illustrating immunodominant *Mtb* antigens that have conserved T-cell epitopes induced a predominant Th1 response, while rare *Mtb* antigens with variable T-cell epitopes were skewed toward Th17 responses. Overall, Ogongo highlighted that distinct *Mtb* antigens are associated with infection outcomes, induce functional responses that precede progression to TB disease, and can determine human T-cell differentiation.

Next, Sam Behar (University of Massachusetts Chan Medical School), highlighted evidence suggesting that poor T-cell recognition of infected cells is a barrier to protective immunity. T-cell recognition of *Mtb*-infected cells may depend on the number of bacteria or the amount of antigen present (3), and further evidence indicates murine *Mtb-*specific CD8 T-cells only recognize infected macrophages with high bacterial burden (4). Thus, high intracellular numbers of *Mtb* may induce macrophage death, release of bacterial antigens or exosome production, leading to acquisition of antigens and cellular debris by uninfected bystander cells that can cross-present antigens. It is therefore important to understand how antigen presentation by bystander antigen presenting cells (APC) and the consequent T-cell activation affect disease pathogenesis.

Cecilia Lindestam Arlehamn (La Jolla Institute for Immunology), finally highlighted differentially recognized T-cell epitopes in the spectrum of *Mtb* infection. Lindestam Arlehamn et al. (5) identified ~400 CD4 T-cell epitopes in IGRA^+^ individuals, with vast heterogeneity of epitope-specific responses to *Mtb*. Recently, Panda et al. (6) provided evidence for differences in T-cell reactivity against *Mtb* antigens between TB active and IGRA^+^ individuals. Using longitudinal cohorts and sequential sampling, future investigations should determine when T-cells begin to respond to these differentially recognized peptide antigens and potentially identify signatures of progression and correlates of protection. The anatomical location of differentially recognized antigens, species specificity, and differences in the antigenic repertoire within an individual are unknown. Furthermore, HLA diversity as a source of heterogeneity should be investigated to identify HLA alleles that are associated with TB disease or contained *Mtb* infection.

During the panel discussion, it was appreciated that TB vaccines are largely based on secreted protein antigens. It was suggested that secreted *Mtb* antigens are likely processed via class II and class I pathways, although only some of these antigens are preferentially taken up by bystander cells. Since certain antigens are more likely to be presented or secreted by an infected cell, multiple antigens in TB vaccines are necessary to confer protective immunity. The tissue microenvironments were also discussed as important for shaping immune responses with regard to antigen presentation and recognition. The granuloma has different microenvironments with cells in different immune states that influence patterns of antigen expression, which ultimately need to be considered and further explored *in vivo*. Since the field lacks a comprehensive understanding of where *Mtb* persists in humans, consideration of alternate concepts of where *Mtb* persists (anatomic and cellular locations) has important implications for vaccine design and delivery. Consequently, mechanisms of extracellular bacteria killing via cytotoxic effectors need consideration. Together, the complexities of tissue microenvironments, *Mtb* strain diversity, and *Mtb* location remain bottlenecks to experimentally evaluate antigen recognition *in vivo*.

### 1.2 Immune control of intracellular infection

The second session focused on immune control of intracellular infection. Mihai Netea (Radboud University Medical Center) presented on BCG-induced trained immunity that elicits an antigen-agnostic response through mechanisms involving immunological pathways, metabolic rewiring, and epigenetic processes in responding cells. BCG-induced control of *Mtb* infection is associated with enhanced myeloid- and innate-associated responses (7). Regarding trained immunity, Moorlag et al. (8) showed that administration of β-glucan prior to *Mtb* infection induced higher functional responses by monocytes *in vitro*, reduced bacterial burden *in vitro* and *in vivo*, and conferred longer survival in infected mice. During the panel discussion, it was noted that the superior efficacy of intravenous BCG (9–11) may be due to the effect of the vaccine on bone marrow progenitor cells (central trained immunity). Importantly, trained immunity involves innate cells beyond myeloid subsets, including non-conventional T-cells, NK cells, and other innate lymphoid cells (ILCs). Trained immunity is improved when there is interaction with the cells of the adaptive immune system which secrete IFN-γ to amplify trained immunity. The panel noted that natural mucosal immunity demonstrates “tolerance-associated” features. For example, lung NK cells generally lack evidence of activation as a protective mechanism to prevent inflammation-induced pathology [reviewed in Cong and Wei (12)]. Therefore, TB vaccine research should consider harnessing the combined induction of classical adaptive immunity and trained immunity in novel vaccination strategies to elicit responses distinct from mechanisms favoring tolerance in the tissues.

The second speaker, David Lewinsohn (Oregon Health and Science University), presented data and efforts aimed at addressing a fundamental: Where is *Mtb* located in the human host? Lewinsohn proposed that studies should consider vaccine-induced responses in the airways, since favorable airway immunity may provide early control and facilitate the development of adaptive immunity (13). Non-classical, MR1-restricted, *Mtb-*reactive CD8+ T-cells (mucosal-associated invariant T-cells - MAIT) were enriched in the upper airways and efficiently enhanced IFN-γ production in response to *Mtb-*infected epithelial cells (14), suggesting that although epithelial cells are generally poorly infected by *Mtb*, they may provide a niche for *Mtb* persistence. Lewinsohn also presented recent evidence demonstrating that T-cell functionality, rather than phenotype, is associated with *Mtb* restriction in the non-human primates (NHP) granuloma (15), and polycytotoxic T-cells can control *Mtb* (16–18). Thus, new vaccines and the analysis of vaccine responses should consider the cytolytic capacity of vaccine-induced responder cells and the cytolytic molecules that restrict *Mtb* growth.

Finally, Henry Mwandumba (Malawi Liverpool Wellcome Programme), focused on how innate immune cells in the human lung shape responses to *Mtb* infection and explored reprogramming of the lung myeloid compartment to prevent the establishment of infection. Boosting early innate responses in the lung may be necessary to limit *Mtb* growth and clear initial infection (19). Upon *Mtb* exposure, different myeloid cell populations in the lung are infected by *Mtb* (20, 21), some of which are effective at controlling infection, while others provide a niche for *Mtb* persistence. However, permissive cells contributing toward the establishment of infection are not well characterized and there is limited data on bacterial permissiveness as an immune function. There is a need for identifying correlates of bacterial permissiveness, in addition to immune correlates of protection and/or risk. Mwandumba then discussed macrophage ontogeny as the primary underpinning to macrophage-associated control of *Mtb* infection in the lung, as demonstrated by Huang and colleagues (21). Specifically, the ability to control *Mtb* infection was determined by the metabolic profile of macrophages: glycolysis predominated in the restrictive recruited interstitial macrophages, while fatty acid oxidation was the dominant metabolic pathway of permissive alveolar macrophages in the murine lung. Thus, the evidence suggests that epigenetic programming and metabolic rewiring of tissue-resident macrophage lineages by new TB vaccines could be a novel strategy to impact *Mtb* infection, control, and persistence in humans.

Relevent to TB immunity in the lung, it was recently (22) demonstrated that tissue-specific activation of T-cells influences their effector functions. Specifically, IFN-γ in the mediastinal lymph node skewed T-cells to be Th1-like effector regulatory T-cells driving their close interaction with type 1 dendritic cells (DCs) and subsequent suppression of cytotoxic T-cell responses. It is worth considering whether a similar mechanism applies during TB. Studying the lung airways provides an opportunity to understand early events occurring upon exposure to the bacteria. To address the limitation of obtaining human lung tissue samples, it is possible to leverage computational approaches to infer the interactions in the alveolar space to the lung parenchyma.

### 1.3 Putting antigen recognition in a clinical context

The third session fostered discussions on translating basic findings into clinical settings. Stephen Carpenter (Case Western Reserve University) highlighted the possible causes and mechanisms of delayed memory T-cell responses to infected cells in the lung. T-cells are detected in the lungs ~2 weeks following aerosol infection (23–25). However, many of the antigen-specific CD4 T-cells in the lungs do not produce IFN-γ following infection (26). Carpenter proposed that there may be a lack of T-cell recognition of infected alveolar macrophages, which accounts for nearly all *Mtb*-infected cells during this initial period (27, 28). *Mtb*-infected macrophages can evade T-cell recognition using Type I and Type II evasion. General inhibition of T-cell recognition (Type I evasion) occurs independent of TCR/antigen specificity and results from inhibitory cytokines or impaired pro-inflammatory cytokine and co-stimulatory receptor expression by *Mtb*-infected macrophages (29, 30). On the other hand, Type II evasion of T-cell recognition is TCR-dependent and/or antigen-specific (31). During Type II evasion, TCR/antigen-specific recognition can be hindered by *Mtb* changing its patterns of antigen expression, antigen export from infected to non-infected cells (32), or altered antigen repertoires presented by dendritic cells (DCs) during T-cell priming, which may be different from peptides presented by infected macrophages in the lung. Indeed, some studies have identified TCRs that were unable to recognize infected macrophages and were limited in their ability to control bacterial infection in mice [reviewed in Yang et al. (33)] and in human vaccination studies (34). Finally, Carpenter presented data from his lab showing that the addition of *Mtb* antigens to *Mtb*-infected macrophages can increase the number of activated human CD4 T-cells in some individuals. Using TCR sequencing, certain clonally-expanded TCRs were identified as unique to the addition of exogenous antigens, providing further evidence of the antigen-specific mechanisms that may govern Type II evasion of T-cell recognition in humans.

Next, Munyaradzi Musvosvi (South African Tuberculosis Vaccine Initiative, University of Cape Town), focused on the use of single-cell TCR sequencing of *Mtb*-specific T-cells to identify priority antigens for TB vaccine development. Bertholet et al. (34) demonstrated that distinct *Mtb* antigens varied in their ability to confer a protective response against *Mtb*. Antigen availability can determine the quality of T-cell response (35). For example, antigens expressed at high levels throughout the course of *Mtb* infection (e.g., ESAT-6) drive terminal differentiation of T-cells, while antigens poorly expressed during the chronic phase of infection (e.g., Ag85B) result in less differentiated T-cells (35). Recently, Musvosvi clustered *Mtb–*specific T-cells into TCR similarity groups based on shared amino acid motifs within CDR3β chains and identified groups enriched in either progressors or controllers, in two longitudinal cohorts (1) (also highlighted by Ogongo in his talk). Further studies using a reporter system to perform *Mtb* genome-wide protein screening (36) identified the cognate epitopes to some of the similarity groups enriched in controllers and progressors, suggesting the existence of “good” and “bad” antigens (37, 38). Musvosvi, therefore, presented a platform for identifying antigens to prioritize for TB vaccine R&D.

Chetan Seshadri (University of Washington) closed the session by focusing on protection from the perspective of the human infection model. Seshadri highlighted the concept of “resistors,” who did not develop evidence of infection or active disease, despite close contact with active TB cases (39), but exhibit immune responses to *Mtb* antigens that were IFN-γ-independent (40). Seshadri then discussed that the “non-conventional” immune response to *Mtb* is mediated, partly, by donor-unrestricted T-cells (DURTs) (41), including MR1-restricted T-cells, which recognize non-peptide antigens (42). Several studies suggest that DURTs may provide help to other immune cells, rather than acting as primary effectors (43–45). Seshadri proposed that antigens recognized by DURTs should be validated and included in pre-clinical and clinical development. For vaccination strategies, it is worth considering whether DURTs can be engineered to induce an effector response and adaptive immunity and whether those responses can be *Mtb*-specific. Seshadri suggested that developing a controlled human *Mtb* infection model could be considered for evaluating *in situ* T-cell clonotypic expansions to discover new antigens for TB vaccines using approaches developed by Munyaradzi and colleagues.

IFN-γ-independent responses to *Mtb* antigens was discussed at length during open forum. While quantifying IFN-γ remains informative and crucial, other cytokine responses to various *Mtb* antigens also provide valuable readouts. Since there is overlap between antigens and antigen specificity, a combination of immune response readouts and inclusion of multiple antigens should be considered in future vaccine studies. The existence of *Mtb* strain-specific transcriptional changes in infected macrophages has been reported (46), and *Mtb* strains are differentially recognized by toll-like receptors (TLRs) with an impact on the immune response (47). Furthermore, human macrophage responses to clinical isolates from *Mtb* complex can discriminate between ancient and modern lineages (48). Thus, it is possible that *Mtb* strain-specific immune responses could inform whether prior exposure favors subsequent immune responses that determine the clinical outcome. The panel discussion also considered the types of antigens used in immunological assays and how, for example, *Mtb* lysates and peptide pools differentially influence the type of cells that respond in recall assays. Therefore, it is imperative to identify signatures that encompass a holistic response and include more immune cells involved in immunity to TB beyond conventional T-cells. Overall, there is a need to invest in systems that integrate various mechanisms for detecting novel antigens and exploring existing antigens, while evaluating the harmonized and interlinked role of immune cells in containing *Mtb* infection.

### 1.4 Developing clinical correlates that reflect recognition of the infected cell

The last session focused on issues regarding the development of clinical correlates that reflect recognition of the infected cell. Bryan Bryson (Massachusetts Institute of Technology) presented FucoID as a technology for identifying antigen-specific T-cells and cell-cell interactions and discussed the potential of this technique as an unbiased approach. With FucoID technology, the fucosyltransferase (FT)-labeled (49) *Mtb-*infected cell acts as a “living tetramer,” which in turn, labels interacting T-cells. Paired with technologies such as CITEseq, FucoID enables phenotypic characterization of *Mtb-*specific T-cells. Potentially all cells interacting with labeled, *Mtb-*infected cells could be identified and used to determine the proportion of vaccine-induced responses. Such tools could also be employed for understanding early immune interactions occurring in the lung airways following *Mtb* exposure.

Simone Joosten (Leiden University Medical Center) presented on growth inhibition assays as a correlate of protection against TB. The Mycobacterial Growth Inhibition Assay (MGIA) provides a measure of growth control by immune cells and gives insights into mechanisms associated with protective immune responses against *Mtb* infection (7). New data from Joosten's group (*in revision*), found that MGIA identified groups of individuals who could or could not control growth of BCG pre- or post-vaccination. Moreover, mechanisms of control may be different between natural and vaccine-induced protection. Since functional measurements of effector responses are important to evaluate protective immunity, improvement of the MGIA platform will provide novel insights into host defense mechanisms associated with control.

The last speaker of this session was Jacqueline Achkar (Albert Einstein College of Medicine) who spoke on protective antigen specificity of antibodies and B cells in TB. Several studies have provided clear evidence for antibody-mediated immunity against TB, including FcγR-mediated effects but knowledge on protective B cell antigens remains limited (50–56). Recent mucosal and intravenous BCG vaccine studies with NHPs showed associations of airway IgA, IgG, and IgM with protection against TB disease (9, 57, 58), highlighting the potential protective role of antibodies and B cells in the lung during initial *Mtb* exposure and infection [reviewed in Boom et al. (59)]. Ishida and colleagues (Achkar lab, in press) have identified IgA responses to epitopes of the *Mtb* surface glycan arabinomannan prior to *Mtb* infection that were associated with the outcome of controlled (LTBI) vs. TB disease outcome in cynomolgus macaques, suggesting that pre-existing antibodies can protect against TB. However, despite the evidence for a protective role of antibodies and B cells in TB, major knowledge gaps, such as the following, remain: (i) The range of protective *Mtb* antigens and their epitopes, including their expression on infected cells; (ii) Differences between mucosal and systemic antibodies and their mechanisms of protection; (iii) The role of antibody isotypes at different stages of *Mtb* infection; and (iv) The role of B cells, independent of antibodies, and their interaction with other immune cells. During the panel discussion, the role of antibodies was discussed in terms of their contributions toward the formation of immune complexes that trigger complement activation, which has been identified as a correlate of risk in TB progressors and TB patients. However, it remains to be determined which specific antibody:antigen complexes contribute toward complement activation and their associations with disease outcome. Finally, exposure to non-tuberculous mycobacteria (NTM) and *Mtb* strains with different virulence and expressed antigenic repertoires were discussed as potential contributing factors toward the heterogeneous effects of B cell and antibody responses.

## 2 Conclusions

A comprehensive understanding of immune recognition and control of the *Mtb-*infected cell is required to accelerate vaccine development against TB. There is no consensus on the definition of a functional profile that infers a protective immune response to *Mtb* in humans. Furthermore, whether a functional profile is determined by the host or driven by the *Mtb* antigens themselves, or both is unresolved. These issues are relevant for vaccine design, where immunogenic antigens are intended to induce protective (long-lasting) immunity. However, in the absence of a functional readout reflecting a protective profile in humans, it is challenging to infer which antigens are ideal for inclusion in novel vaccine candidates. We propose that investing more research into developing correlates of protection would be greatly beneficial and impactful. We also propose that vaccine trials include an array of readouts, in addition to efficacy and T-cell immunogenicity endpoints. For example, bacterial killing to evaluate bacilli clearance by effector cells and quantifying functional responses beyond conventional cytokines and T-cells. The use of spatial technologies holds potential for identifying vaccines with protective efficacy for development of clinical correlates reflecting recognition of the infected cell. A major theme of the workshop was to explore T-cell subsets beyond CD4 T-cell recognition. In this regard, other T-cell subsets, such as CD8 T-cells and DURTs, require more investigation into their antigen recognition capabilities to discover novel antigens for inclusion as potential vaccines.

Human TB research relies on studying immune responses in circulation by flow-based assays. While valuable, these studies are limited by their inability to mirror the immune responses at the site of disease. Furthermore, the knowledge of the epitopes displayed by *Mtb*-infected cell is limited, thus, there is a need for better measurements of these responses. Sampling broncho-alveolar lavage (BAL) fluid enables studying TB immunology in the airways and alveolar space but interactions in the airways do not fully represent the lung parenchyma. Although sampling human tissues is not possible in a clinical setting, postmortem tissue sampling offers alternatives. Multi-omics approaches in humans and NHPs hold great potential to develop tools that accurately infer interactions and responses to bridge the gap between the periphery, airways, and parenchyma in humans. Finally, since well-characterized human cohorts are fundamental to vaccine research, studies that include overlap between different stages of infection and disease are important to capture the spectrum of TB disease, and such studies should integrate clinical data to capture the variability between cohorts and participants. Additional studies should consider development of controlled human infection models to provide insights into variability observed in *Mtb* infection outcomes. Thus, the key bottlenecks in advancing TB vaccine research are the absence of immune correlates of protection for validation of vaccine candidates in development and the lack of knowledge of where Mtb is located the human host. However, opportunities exist to accelerate efforts to develop better TB vaccines through computational approaches that enable projection of observations in animal models into humans and the use of multi-species approach to identify correlates of protection.

## Author contributions

CY: Writing – original draft, Writing – review & editing. MNM: Writing – original draft, Writing – review & editing. PO: Writing – original draft, Writing – review & editing.
